# Managing Needs-based Inpatient Care for Young Adults: Project ‘House of the Future’

**DOI:** 10.1192/j.eurpsy.2025.1560

**Published:** 2025-08-26

**Authors:** U. Altunoez, A.-C. Hergott, I. B. Guevercin, S. Akbas, A. Daskiewicz, L. Schmidbauer, C. Dette

**Affiliations:** 1 Department of General Psychiatry & Psychotherapy, KRH Wunstorf Psychiatric Hospital, Wunstorf; 2 Research Group for Social and Transcultural Psychiatry and Psychotherapy, Hannover Medical School, Hannover, Germany

## Abstract

**Introduction:**

The transition from childhood to adulthood is a complex process and an important developmental task with many opportunities and risks. Despite the fact that almost three-quarters of psychiatric disorders begin before the age of twenty-four, adolescents and young adults are poorly represented in psychiatric research, and the data used to guide diagnosis and treatment in this group rely mainly on studies in adult populations. The mental health system in Germany is also not organized to meet the needs of this age group, and as a result, the treatment outcomes for young adults in inpatient or day-care units of adult psychiatric centers are mostly unsatisfactory. For this reason, it is crucial to implement mental health care concepts that are tailored to the needs of young adults.

**Objectives:**

Here we present a milieu-therapeutic inpatient care concept, ‘House of the Future,’ which integrates different therapeutic approaches to provide holistic and targeted support for young adults in need of psychiatric care.

**Methods:**

An innovative inpatient psychiatric care concept for young adults from a psychiatric training hospital in Lower Saxony, Germany, will be presented in detail.

**Results:**

The concept of the ward incorporates a variety of therapeutic approaches, including milieu therapy, systemic family therapy, and psychodynamic therapy. Additionally, it incorporates safe-wards elements and numerous non-verbal, innovative techniques such as art therapy, occupational therapy, music therapy, and sports therapy adapted for this age group. We provide a structured and needs-based environment for young adults, enabling them to engage with their experiences without being defined by their disorder or solely integrated into psychiatric systems. Our holistic approach aims not only to treat symptoms but also to promote sustainable personal development and a healthy lifestyle that empowers young adults. Young adults represent the future, and the development of their future is contingent upon the efficacy of the social support system, which is itself a significant challenge. Consequently, social work and pedagogical support are among the most essential elements of the concept. In addition to the comprehensive presentation of the concept, opportunities for networking with integration support institutions, day clinics, and specific outpatient units will be provided.

**Conclusions:**

Adolescence and young adulthood are distinct developmental stages with unique needs. To reach optimal outcomes and to build our future on a safe base, it is fundamental to implement tailored care models in psychiatric settings that are in harmony with the specific requirements of this age group. Project ‘House of the Future’ aims to be a best practice example to address these needs.

**Disclosure of Interest:**

None Declared

